# A Complex Impact of Systemically Administered 5-HT_2A_ Receptor Ligands on Conditioned Fear

**DOI:** 10.1093/ijnp/pyab040

**Published:** 2021-07-06

**Authors:** Sven Melker Hagsäter, Robert Pettersson, Christopher Pettersson, Daniela Atanasovski, Jakob Näslund, Elias Eriksson

**Affiliations:** Department of Pharmacology, Institute of Neuroscience and Physiology, Sahlgrenska Academy, University of Gothenburg, Sweden

**Keywords:** 5-HT2A receptor, MDL 100907, pimavanserin, psilocybin, TCB-2

## Abstract

**Background:**

Though drugs binding to serotonergic 5-HT_2A_ receptors have long been claimed to influence human anxiety, it remains unclear if this receptor subtype is best described as anxiety promoting or anxiety dampening. Whereas conditioned fear expressed as freezing in rats is modified by application of 5-HT_2A_–acting drugs locally into different brain regions, reports on the effect of systemic administration of 5-HT_2A_ receptor agonists and 5-HT_2A_ antagonists or inverse agonists on this behavior remain sparse.

**Methods:**

We assessed the possible impact of systemic administration of 5-HT_2A_ receptor agonists, 5-HT_2A_ receptor inverse agonists, and a selective serotonin reuptake inhibitor (SSRI)—per se or in combination—on the freezing displayed by male rats when re-exposed to a conditioning chamber in which they received foot shocks 7 days earlier.

**Results:**

The 5-HT_2A_ receptor agonists psilocybin and 25CN-NBOH induced a reduction in conditioned fear that was countered by pretreatment with 5-HT_2A_ receptor inverse agonist MDL 100907. While both MDL 100907 and another 5-HT_2A_ receptor inverse agonist, pimavanserin, failed to impact freezing per se, both compounds unmasked a robust fear-reducing effect of an SSRI, escitalopram, which by itself exerted no such effect.

**Conclusions:**

The results indicate that 5-HT_2A_ receptor activation is not a prerequisite for normal conditioned freezing in rats but that this receptor subtype, when selectively over-activated prior to expression, exerts a marked fear-reducing influence. However, in the presence of an SSRI, the 5-HT_2A_ receptor, on the contrary, appears to counter an anti-freezing effect of the enhanced extracellular serotonin levels following reuptake inhibition.

**Significance Statement**

The impact of serotonergic 5-HT_2A_ receptors on mood and anxiety has recently gained enhanced attention. Thus, while adding drugs blocking 5-HT_2A_ receptors to treatment with selective serotonin reuptake inhibitors (SSRIs) has been claimed to augment the antidepressant and anxiety-reducing effect of the latter, also 5-HT_2A_ activation, obtained with psychedelic drugs such as psilocybin, has been attributed a beneficial effect in the treatment of mood disorders. In the present study, experiments addressing the impact of systemic administration of 5-HT_2A_ receptor agonists and 5-HT_2A_ receptor inverse agonists on the expression of context-conditioned fear yielded results similar to those reported from clinical studies. Thus, while 5-HT_2A_ agonists reduced conditioned freezing, 5-HT_2A_ receptor inverse agonists did not impact this response per se but unmasked an anti-freezing effect of an SSRI. The results lend further support for the assumption that pharmacological manipulation of 5-HT_2A_ receptors may impact anxiety.

## Introduction

Compounds displaying affinity for the serotonergic 5-HT_2A_ receptor have long been claimed to impact human anxiety. 5-HT_2A_ receptor agonists, such as the psychedelic compounds psilocybin and lysergic acid diethylamide, may thus induce anxiety (Hofmann, 1959; Barrett et al., 2016; Carbonaro et al., 2016; Carhart-Harris et al., 2016) but have also been attributed long-lasting anxiety-reducing and antidepressant effects (Carhart-Harris and Nutt, 2017; Nutt, 2019; Goldberg et al., 2020). And while selective 5-HT_2A_ receptor antagonists are not anxiogenic and have failed to display convincing efficacy in anxiety disorders when administered per se (Westenberg and den Boer, 1989), 5-HT_2A_ receptor antagonism and inverse agonism have been suggested to augment the antidepressant and anxiety-reducing effect of selective serotonin reuptake inhibitors (SSRIs) (see below).

Conditioned fear expressed as freezing in animals (Fanselow and Helmstetter, 1988; LeDoux, 2014) is frequently used to reflect aspects of human anxiety and appears dependent on an intact serotonergic transmission (Bauer, 2015; Bocchio et al., 2016). An increase in extracellular levels of serotonin in different brain regions has thus been observed during exposure to both an unconditioned (Kawahara et al., 1993; Rueter and Jacobs, 1996; Mitsushima et al., 2006; Fulford and Marsden, 2007; Hassell et al., 2019) and a conditioned (Yoshioka et al., 1995; Hashimoto et al., 1999; Yokoyama et al., 2005; Zanoveli et al., 2009) fear stimulus, and the normal freezing response is impaired both in animals exposed to serotonin depletion (Archer et al., 1982; Inoue et al., 1996a; Pettersson et al., 2016) and in those administered drugs markedly increasing extracellular levels of serotonin (Inoue et al., 1996a; Miyata et al., 2007) prior to expression. The 5-HT_2A_ receptor playing an important role in this context gains support from studies exploring the effect of local modulation of 5-HT_2A_ receptors in different brain regions such as the central (Isosaka et al., 2015) or basolateral (Macedo et al., 2007; Clinard et al., 2015) amygdala, the dorsolateral septum (de Paula et al., 2012), the bed nucleus of the stria terminalis (Hammack et al., 2009), and the dorsal periaqueductal grey (Brandao et al., 2008). However, studies exploring the impact of selective 5-HT_2A_ receptor agonists, antagonists, and inverse agonists on the expression of conditioned fear when systemically administered, that is, as in the clinical situation, are as yet relatively sparse (Inoue et al., 2011; Bauer, 2015).

While SSRIs are first-line treatment for a number of anxiety disorders (Eriksson and Humble, 1990; Nutt, 2005), initially these drugs may, on the contrary, elicit or augment anxiety (Sinclair et al., 2009; Naslund et al., 2017). Similarly, in fear-conditioned rats, both freezing-dampening (Hashimoto et al., 1996; Inoue et al., 1996b; Santos et al., 2006; Montezinho et al., 2010) and freezing-promoting (Burghardt et al., 2007; Montezinho et al., 2010; Pettersson et al., 2015; Hagsater et al., 2019) effects of acute exposure to an SSRIs have been reported. While the reasons for these differences between studies remain obscure, the capability of an SSRI to exert both an anxiogenic-like and an anxiolytic-like influence on the expression of fear-conditioned freezing suggests that the enhanced extracellular levels of serotonin obtained by acute serotonin reuptake inhibition may activate both freezing-promoting and freezing-dampening pathways, the behavioral outcome being dependent on the relative impact of these—and possibly on the relative impact of different postsynaptic receptor subtypes—in the experimental situation at hand. A possible involvement of the 5-HT_2A_ receptor in this context gains support from clinical reports indicating that the efficacy of SSRIs may be augmented by adding compounds selectively (Fava et al., 2019; Papakostas et al., 2020) or non-selectively (Maes et al., 1999; Carpenter et al., 2002; Marek et al., 2003; Corya et al., 2006; Rapaport et al., 2006; Blier et al., 2010; Carey et al., 2012), blocking this receptor subtype or acting as an inverse agonist on it.

The aims of the present study were to explore the possible impact of systemic administration of 5-HT_2A_ receptor agonists on the expression of context-conditioned freezing and also to address the effects of 5-HT_2A_ receptor inverse agonists, administered per se or in combination with either a 5-HT_2A_ receptor agonist or an SSRI, escitalopram, on this behavior. The possible effects of a 5-HT_2A_ receptor inverse agonist and a 5-HT_2C_ receptor antagonist, given alone or with escitalopram, were also investigated.

## Methods

### Animals

A total of 415 male Sprague Dawley rats (Taconic, Borup, Denmark and Janvier, Le Genest-Saint-Isle, France) were used in the fear conditioning experiments and 60 male Wistar Hannover rats (Taconic, Ejby, Denmark) were used in the elevated plus maze (EPM) study. The animals were 9–10 weeks of age on arrival and habituated to human contact until the start of experiments 2–3 weeks later. They were housed 3 per cage in an animal facility where they were maintained on a 12-hour-light/-dark cycle. All experiments had been approved by the Animal Ethics Committee at the University of Gothenburg in accordance with the guidelines of the Swedish Board of Agriculture.

### Context-Conditioned Fear

**Figure 1.**
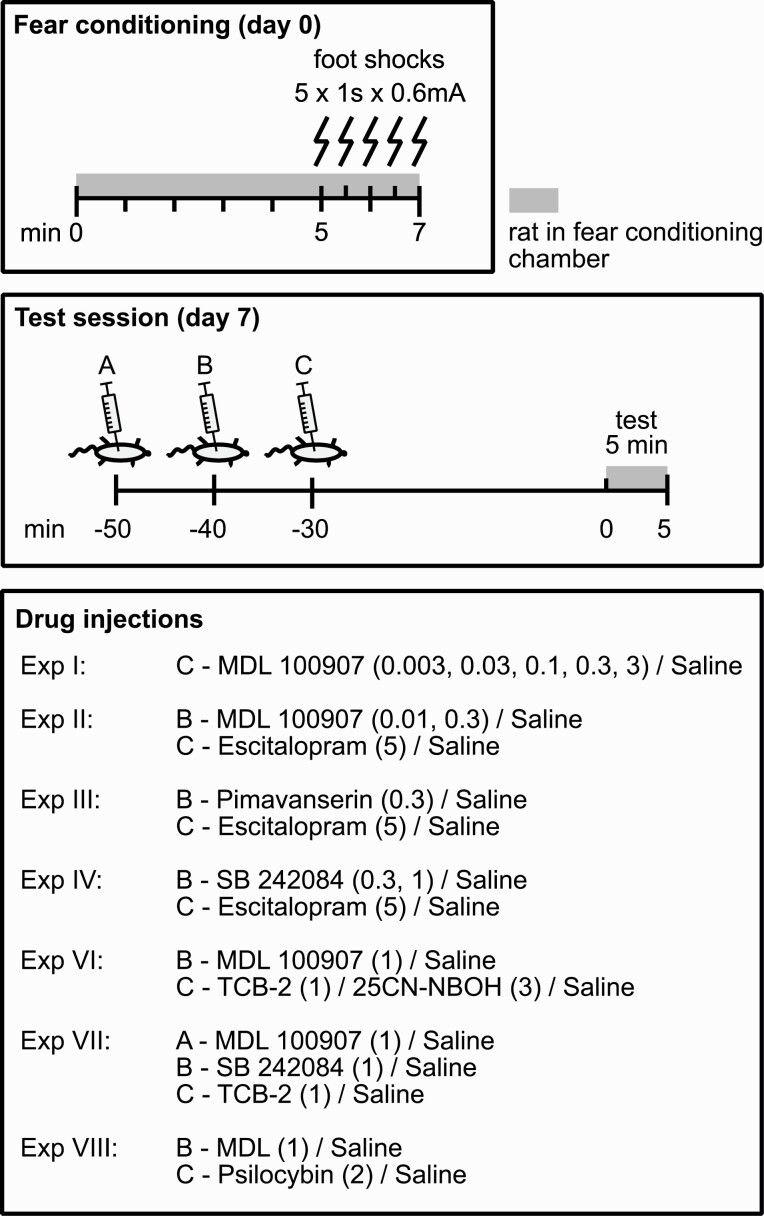
Fear conditioning protocol and time of injections in the fear conditioning experiments (I–IV and VI–VIII). Doses in parentheses are given as mg/kg.

The contextual fear experiments were performed using the Contextual NIR Video Fear Conditioning System for Rat (Med Associates, Saint Albans, VT, USA) consisting of a conditioning chamber with a grid floor through which the rats, after an acclimatization period of 5 minutes, received 5 electric foot shocks (0.6 mA, 30-second inter-shock interval) during a time period of 2 minutes (Figure 1). The chamber was enclosed by a sound-attenuating cubicle in which a constant 60-dB white background noise was delivered during all experimental sessions. Seven days later, the rats were re-exposed to the conditioning chamber for assessment of context-conditioned freezing calculated by automatic scoring of video recordings (immoblity >1 second) and presented as the percentage (%) of the 5-minute test that the animal spent immobile (Anagnostaras et al., 2010; Hagsater et al., 2019). For any rat showing immobility, gross observation was applied to confirm that the animal displayed typical signs of freezing, that is, rigged tail, arched back, erected fur, and retracted ears (Riess, 1945; Brandao et al., 2008). The faeces tray was emptied and the wire mesh cage was cleaned using 70% ethanol before every new test session. Drug injections were administered 50, 40, and/or 30 minutes prior to test.

### Elevated Plus Maze

For the EPM experiment, a standard black acrylic plastic rat EPM (Med Associates) was placed in a quiet room with a single incandescent light bulb providing a light level of 35 lux in the centre of the maze. The animals were moved to the room for acclimatization 90 minutes prior to testing. Drug or saline injections were administered 40 and/or 30 minutes prior to test.

The rats spent 5 minutes in the maze, during which the number of entries onto open arms, and the total time spent on open arms was captured using vido recordings and manually analyzed after the experiment.

### Drugs

MDL 100907, TCB-2, and 25CN-NBOH were purchased from Tocris (Bristol, UK), escitalopram oxalate was purchased from Shodana Labs (Hyderabad, India), pimavanserin was purchased from MedChem Express (Monmouth Junction, NJ, USA), psilocybin was purchased from Chiron Corporation (Emeryville, CA, USA), and SB 242084 was purchased from Sigma-Aldrich (St. Louis, MO, USA). Escitalopram and 25CN-NBOH were dissolved in 0.9% saline. SB 242084 was dissolved in 0.9% saline, 8% cyclodextrin, and 0.48% citric acid. All other drugs were dissolved in 0.9% saline by adding a small volume of 1 M HCl while the solution was heated and stirred. When required, pH was readjusted by adding NaOH. Controls received 0.9% saline. All injections were given subcutaneously with an injection volume of approximately 1 mL. For time points of injections, see Figure 1.

### Statistics

The data were analyzed using SPSS version 19. Comparison of groups were undertaken using Kruskal-Wallis tests followed by Mann-Whitney *U* tests.

## Results

### Effect of the 5-HT_2A_ Receptor Inverse Agonist MDL 100907 on the Expression of Conditioned Fear (Experiment I)

**Figure 2.**
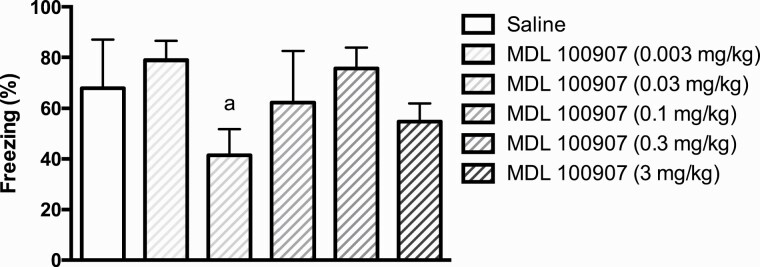
Effect of the 5-HT_2A_ receptor inverse agonist MDL 100907 on conditioned freezing displayed as percentage of time. MDL 100907 was administered 30 minutes prior to testing. Number of animals per group = 12. Bars represent median time spent freezing ± interquartile range. a = *P* < .05 vs saline.

While there was no signficant effect on fear-conditioned freezing of the inverse 5-HT_2A_ receptor agonist MDL 100907 administered per se at doses of 0.003, 0.1, 0.3, and 3 mg/kg, animals administered 0.03 mg/kg displayed moderately reduced freezing (*U* = 35.0, *P* < .05) (Figure 2). In a subsequent experiment, no effect of this dose of MDL 100907 could be confirmed (data not shown).

### Effect of the Combination of the 5-HT_2A_ Receptor Inverse Agonist MDL 100907 and the SSRI Escitalopram on the Expression of Conditioned Fear (Experiment II)

**Figure 3.**
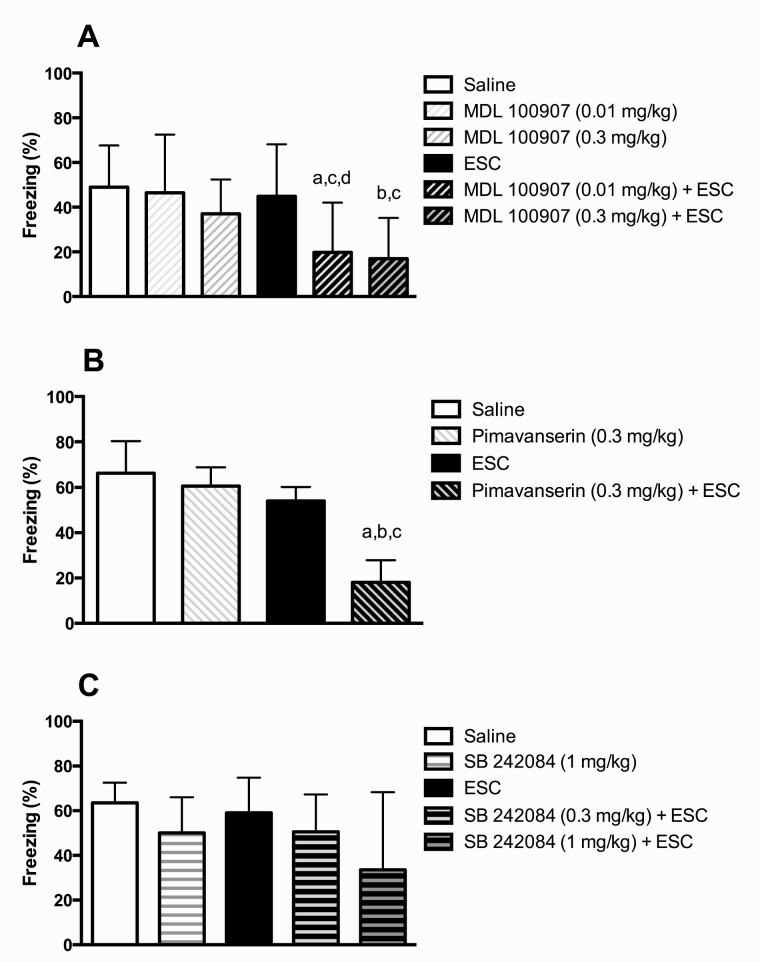
Effect of the selective serotonin reuptake inhibitor escitalopram and different pretreatments (MDL 100907, pimavanserin, and SB 242084) on conditioned freezing displayed as percentage of time. The dose of escitalopram (ESC) was 5 mg/kg in all 3 experiments. MDL 100907, pimavanserin, and SB 242084 were administered 40 minutes prior to testing; escitalopram was administered 30 minutes prior to testing. Bars represent medians ± interquartile range. (A) Number of animals per group = 10. a = *P* < .05 vs saline; b = *P* < .01 vs saline; c = *P* < .01 vs escitalopram; d = *P* < .05 vs MDL 100907. (B) Number of animals per group = 11–12. a = *P* < .001 vs saline; b = *P* < .01 vs escitalopram; c = *P* < .01 vs pimavanserin. (C) Number of animals per group = 12. There were no significant differences between groups.

While neither the SSRI escitalopram (5 mg/kg) nor the inverse 5-HT_2A_ receptor agonist MDL 100907 (0.01 and 0.3 mg/kg) administered per se significantly influenced fear-conditioned freezing, the combination of escitalopram (5 mg/kg) and either dose of MDL 100907 caused a significant reduction compared with groups treated with saline (0.01 mg/kg: *U* = 18.0, *P* < .05; 0.3 mg/kg: *U* = 13.0, *P* < .01), escitalopram (0.01 mg/kg: *U* = 21.0, *P* < .05; 0.3 mg/kg: *U* = 16.0, *P* < .05), and MDL 100907 (0.01 mg/kg: *U* = 16.0, *P* < .05; 0.3 mg/kg: *U* = 26.0, *P* = .07) (Figure 3A).

### Effect of the Combination of the 5-HT_2A_ Receptor Inverse Agonist Pimavanserin and the SSRI Escitalopram on the Expression of Conditioned Fear (Experiment III)

While neither the 5-HT_2A_ receptor inverse agonist pimavanserin (0.3 mg/kg) nor the SSRI escitalopram (5 mg/kg) administered per se significantly influenced fear-conditioned freezing, the combination of escitalopram (5 mg/kg) and pimavanserin caused a significant reduction compared with groups treated with saline (*U* = 1.5 *P* < .001), escitalopram (*U* = 19.0, *P* < .01), and pimavanserin (*U* = 13.0, *P* < .01) (Figure 3B).

### Effect of the Combination of the 5-HT_2C_ Receptor Antagonist SB 242084 and the SSRI Escitalopram on the Expression of Conditioned Fear (Experiment IV)

None of the following treatments influenced fear-conditioned freezing: (1) the SSRI escitalopram (5 mg/kg); (2) the 5-HT_2C_ receptor antagonist SB 242084 (1 mg/kg); (3) escitalopram (5 mg/kg) plus SB 242084 (0.3 mg/kg); or (4) escitalopram (5 mg/kg) plus SB 242084 (1 mg/kg) (Figure 3C).

### Effect of the Combination of the 5-HT_2A_ Receptor Inverse Agonist MDL 100907 and the SSRI Escitalopram on Time Spent on and Entries Onto Open Arms in the EPM (Experiment V)

**Figure 4.**
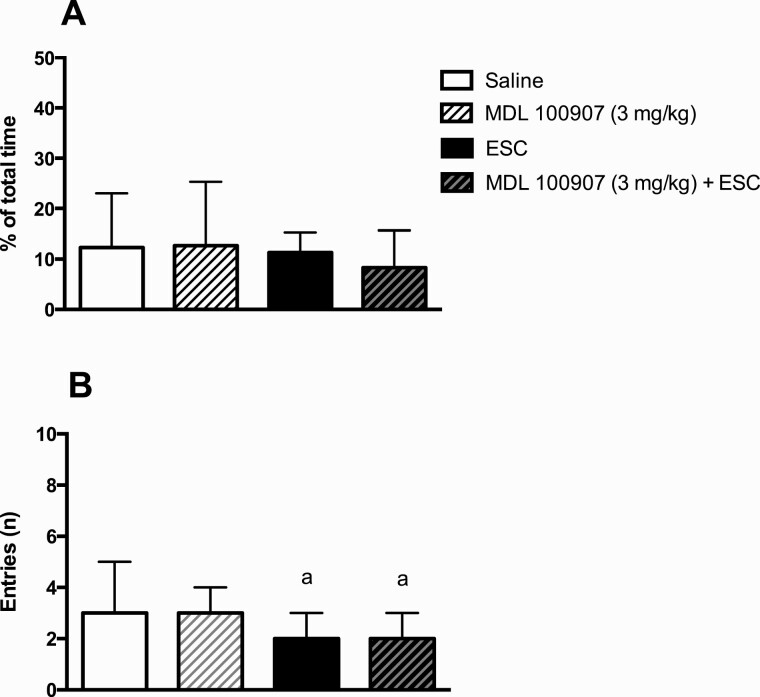
Effect of the selective serotonin reuptake inhibitor escitalopram and the 5-HT_2A_ receptor inverse agonist MDL 100907 on behavior in the elevated plus maze. Shown are percentage of total time spent on open arms (A) and number of entries onto open arms (B). The dose of escitalopram (ESC) was 5 mg/kg. MDL 100907 was administared 40 minutes prior to testing; escitalopram was administered 30 minutes prior to testing. Number of animals per group = 15. Bars represent medians ±interquartile range. a = *P* < .05 vs saline.

The SSRI escitalopram (5 mg/kg) did not impact the time spent on open arms (Figure 4A) but reduced the number of entries onto open arms (*U* = 66.0, *P* < .05) (Figure 4B) in the EPM. The inverse 5-HT_2A_ receptor agonist MDL 100907 (3 mg/kg) displayed no effect per se and did not impact the response to escitalopram.

### Effect of the 5-HT_2A_ Receptor Agonists 25CN-NBOH and TCB-2 With or Without Pretreatment With the 5-HT_2A_ Receptor Inverse Agonist MDL 100907 on the Expression of Conditioned Fear (Experiment VI)

**Figure 5.**
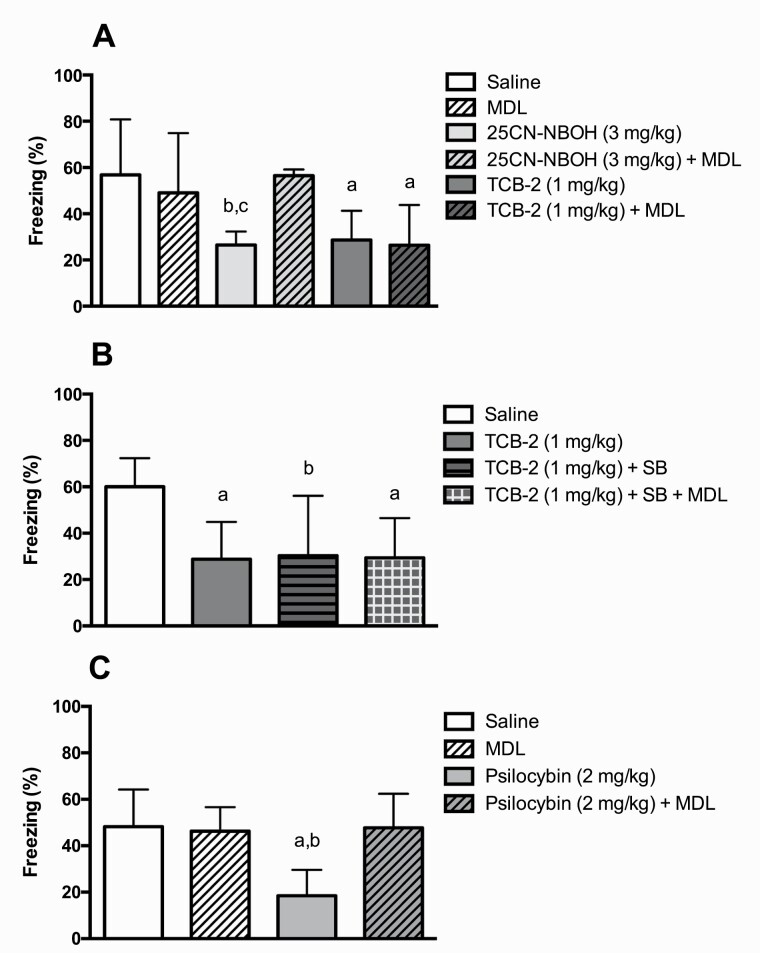
Effect of the the 5-HT_2A_ receptor agonists 25CN-NBOH, TCB-2, and psilocybin with or without pretreatment with the 5-HT_2A_ receptor inverse agonist MDL 100907 and the 5-HT_2C_ receptor antagonist SB 242084 on conditioned freezing displayed as percentage of time. The doses of MDL 100907 (MDL) and SB 242084 (SB) were 1 mg/kg in all experiments. MDL 100907 (A and C), SB 242084 (B), and the combination of the two (B) were administered 40 minutes prior to testing; 25CN-NBOH (A), TCB-2 (A and B), and psilocybin (C) were administered 30 minutes prior to testing. Bars represent medians ± interquartile range. (A) Number of animals per group = 10. a = *P* < .05 vs saline; b = *P* < .01 vs saline; c = *P* < .001 vs 25CN-NBOH plus MDL 100907. (B) Number of animals per group = 7–9. a = *P* < .01 vs saline; b = *P* < .05 vs saline. (C) Number of animals per group = 12. a = *P* < .01 vs saline; b = *P* < .01 vs MDL 100907 plus psilocybin.

Both 5-HT_2A_ receptor agonists 25CN-NBOH (3 mg/kg) (*U* = 13.0, *P* < .01) and TCB-2 (1 mg/kg) (*U* = 21.0, *P* < .05) reduced freezing (Figure 5A). While the effect excerted by 25CN-NBOH was blocked by co-administration with the inverse 5-HT_2A_ receptor agonist MDL 100907 (1 mg/kg) (combination vs 25CN-NBOH: *U* = 6.0, *P* < .001; combination vs saline: *U* = 47.0, *P* = .82), this was not the case for TCB-2 (combination vs TCB-2: *U* = 42.0, *P* = .55; combination vs saline: *U* = 22.0, *P* < .05).

### Effect of the 5-HT_2A_ Receptor Agonist TCB-2 With or Without Pretreatment With the 5-HT_2C_ Receptor Antagonist SB 242084 and the 5-HT_2A_ Receptor Inverse Agonist MDL 100907 on the Expression of Conditioned Fear (Experiment VII)

The freezing-reducing effect of TCB-2 (1 mg/kg) (*U* = 11.0, *P* < .01) was reversed neither by pretreatment with the 5-HT_2C_ receptor antagonist SB 242084 (1 mg/kg) (combination vs TCB-2: *U* = 28.0, *P* = .71; combination vs saline: *U = *9.0, *P* < .05) nor by pretreatment with the combination of SB 242084 (1 mg/kg) and the inverse 5-HT_2A_ receptor agonist MDL 100907 (1 mg/kg) (combination vs TCB-2: *U* = 26.0, *P* = .56; combination vs saline: *U = *5.0, *P* < .01) (Figure 5B).

### Effect of the 5-HT_2A_ Receptor Agonist Psilocybin With or Without Pretreatment With the 5-HT_2A_ Receptor Inverse Agonist MDL 100907 on the Expression of Conditioned Fear (Experiment VIII)

Psilocybin (2 mg/kg) reduced freezing (*U* = 21.0, *P* < .01) (Figure 5C). The effect was reversed by pretreatment with the inverse 5-HT_2A_ receptor agonist MDL 100907 (1 mg/kg) (combination vs psilocybin: *U* = 23.0, *P* < .01; combination vs saline: *U = *71.0, *P* = .96).

## Discussion

The present study is, to our knowledge, the first to demonstrate an anxiety-reducing effect of psilocybin in the conditioned-fear paradigm and also the first to report on a synergistic effect of an SSRI and a 5-HT_2A_ receptor inverse agonist in this model; both these observations are partly in line with clinical observations. Of note is that the results reveal 3 entirely different responses to inverse agonism at 5-HT_2A_ receptors with respect to the expression of conditioned fear: while inverse agonism per se did not impact this response, it opposed an anti-freezing effect displayed by 5-HT_2A_ receptor agonists and, on the other hand, unmasked an anti-freezing effect of an SSRI.

Although previous studies have suggested the 5-HT_2A_ receptor to be critically involved in conditioned fear (Graeff et al., 1996; Weisstaub et al., 2006; Macedo et al., 2007; de Paula et al., 2012; Clinard et al., 2015; Isosaka et al., 2015), the observation that the 5-HT_2A_ receptor inverse agonists MDL 100907 and pimavanserin displayed no consistent effect on the freezing response when administered alone suggests that 5-HT_2A_ receptor activation is not a prerequisite for a normal context-conditioned fear response to take place (Inoue et al., 2011). These observations are in line with a previous report showing no change in context-conditioned freezing in mice genetically depleted of 5-HT_2A_ receptors (Weisstaub et al., 2006).

While we hence observed no impact of 5-HT_2A_ receptor inverse agonists per se on conditioned freezing, it should be underlined that the lack of such an effect in normal experimental rats does not preclude that 5-HT_2A_ receptor antagonism or inverse agonism may be beneficial for the treatment of pathological anxiety in case anxiety disorders—as have been suggested (Esler et al., 2007; Frick et al., 2015)—are characterized by an abnormal increase in serotonergic transmission. Of interest in this context is the notion that 5-HT_2A_ receptors, displaying relatively low affinity for serotonin, may be activated by the endogenous transmitter only when the extracellular levels of this transmitter are high (Carhart-Harris et al., 2016) as well as a previous report showing systemic administration of a non-selective 5-HT_2A_ receptor antagonist to reduce conditioned freezing in rats bred to display enhanced freezing while exerting the opposite effect in low-freezing animals (Leon et al., 2017). On the other hand, the present observation that selective 5-HT_2A_ receptor inverse agonists do not modify conditioned freezing is well in line with the dearth of clinical studies convincingly showing such molecules to exert an anxiety-reducing effect per se (Westenberg and den Boer, 1989).

When using the present experimental protocol, escitalopram at a dose of 5 mg/kg administered per se neither augmented nor reduced conditioned fear; the same was also true for lower and higher doses of the drug (Hagsäter SM, Eriksson E). However, when low doses of either MDL 100907 or pimavanserin were administered prior to escitalopram, neither of which influenced freezing when given alone, a marked anti-freezing response was observed. It is tempting to relate this observation to recent clinical reports indicating that pimavanserin exerts an anxiety-reducing effect when added to an SSRI in patients with anxious depression (Fava et al., 2019; Papakostas et al., 2020), and it may also be of relevance for the beneficial effects attributed to atypical antipsychotic agents as well as the antidepressant mirtazapine—all of which are 5-HT_2A_ receptor antagonists—as an SSRI add-on (Marek et al., 2003). Of note is, however, that a synergy between a 5-HT_2A_ receptor antagonist and an SSRI does not seem to be at hand in all animal models of anxiety. Thus, in the EPM experiment, escitalopram exerted an anxiogenic effect on entries onto open arms, which is in line with previous studies (Naslund et al., 2015), that was neither countered nor reversed by pretreatment with MDL 100907. On the other hand, a synergistic effect of 5-HT_2A_ receptor antagonism and serotonin reuptake inhibition has previously been reported in animal models of depression (Marek et al., 2005; Quesseveur et al., 2013).

A possible explanation for the observed synergy between escitalopram and 5-HT_2A_ receptor inverse agonists on conditioned fear would be that the increase in extracellular levels observed in some (but not all) brain areas following acute SSRI administration (Fuller, 1994; Fritze et al., 2017) activates both anxiety-promoting and anxiety-dampening serotonin receptor subtypes, the net impact being nil, and that 5-HT_2A_ receptors in this situation exert an anxiety-provoking influence so that a 5-HT_2A_ receptor antagonist or inverse agonist unmasks an anxiety-reducing influence exerted by other serotonin receptor subtypes. 5-HT_2A_ receptors enhancing conditioned fear have indeed been identified in the basal (Macedo et al., 2007) and the central nucleus of the amygdala (Isosaka et al., 2015) as well as in the bed nucleus of the stria terminalis (Hammack et al., 2009). Alternatively, however, it may be speculated that MDL 100907 and pimavanserin reveal an anti-freezing influence of the SSRI by augmenting its impact on extracellular serotonin so that a level sufficient to counteract freezing is obtained. Lending some support to this possibility, 5-HT_2A_ receptors situated in the prefrontal cortex have been shown to exert a negative feed-back influence on the firing of serotonergic neurons (Boothman et al., 2003; Quesseveur et al., 2013), and MDL 100907 has been reported to augment the influence of an SSRI on extracellular levels of serotonin in the hippocampus (Boothman et al., 2006) (but not in the prefrontal cortex) (Quesseveur et al., 2013).

A previous study suggesting a selective 5-HT_2C_ receptor antagonist, SB 242084, countered an SSRI-induced increase in cue-conditioned fear (Burghardt et al., 2007) prompted us to explore the possible impact of this molecule and escitalopram given in combination. However, unlike MDL 100907 and pimavanserin, SB 242084—administered at the same dose as in the above-mentioned study—did not unmask an anti-freezing effect of the SSRI. Since 5-HT_2C_ receptor antagonists, such as SB 242084, have also been reported to augment the influence of an SSRI on extracellular levels of serotonin (Boothman et al., 2006; Sotty et al., 2009), this observation would indirectly argue against the hypothesis presented in the previous paragraph that the interaction between escitalopram and the 5-HT_2A_ receptor inverse agonists be presynaptically mediated. Of note is that SB 242084 given alone also did not modulate conditioned freezing; under the present experimental conditions, this response in otherwise untreated animals hence appears independent of both 5-HT_2A_ and 5-HT_2C_ receptors.

While a single dose of an SSRI does not exert anxiety-reducing effects in humans, repeated administration is highly beneficial in several different anxiety disorders (Eriksson and Humble, 1990; Nutt, 2005). Similarly, in contrast to acute administration, we have found subchronic SSRI treatment to reduce context-conditioned fear in experiments using the same settings as those presented in this paper (Hagsater et al., 2016). Given the present observation that 5-HT_2A_ receptor inverse agonists unmask an anti-anxiety effect of an SSRI, it is tempting to suggest that a gradual downregulation of postsynaptic 5-HT_2A_ receptors or structures associated with these (Gray and Roth, 2001) may contribute to the sluggish anti-anxiety effect exerted by the SSRIs both clinically and in the rodent-conditioned fear paradigm.

Psychedelic 5-HT_2A_ receptor agonists such as psilocybin have gained widespread attention because of an alleged long-lasting anti-anxiety and antidepressant effect following single doses (Carhart-Harris et al., 2016; Nutt, 2019; Goldberg et al., 2020). The marked freezing-reducing effects induced by both psilocybin and another 5-HT_2A_ agonist, 25CN-NBOH (Hansen et al., 2014), both of which were antagonized by MDL 100907, are well in line with this possibility. However, the active psilocybin metabolite psilocin is an agonist at several other 5-HT receptors as well, which may be important for the therapeutic effect of the drug (Passie et al., 2002).

A third 5-HT_2A_ receptor agonist, TCB-2 (McLean et al., 2006), also reduced conditioned freezing, but this effect was not countered by MDL 100907. Since it has been suggested that TCB-2 may also activate 5-HT_2c_ receptors (Di Giovanni and De Deurwaerdere, 2018), we explored if its effect on conditioned fear could be countered by SB 242084 or by the combination of SB 242084 and MDL 100907, but found this not to be the case. Hence, neither 5-HT_2A_ receptors nor 5-HT_2C_ receptors seem involved in the anti-freezing effect of this molecule.

The freezing-reducing impact of psilocybin and 25CN-NBOH, suggesting the main influence of 5-HT_2A_ receptors in the studied model to be fear-reducing, contrasts with the outcome of the experiment where escitalopram was combined with MDL 100907 and also with previous studies portraying 5-HT_2A_-receptors in the amygdala and the bed nucleus of the stria terminalis as fear-promoting (Graeff et al., 1996; Weisstaub et al., 2006; Macedo et al., 2007; de Paula et al., 2012; Clinard et al., 2015; Isosaka et al., 2015). A possible localization of a 5-HT_2A_ receptor exerting the opposite effect, however, would be the periaqueductal grey, where stimulation of 5-HT_2A_ receptors on inhibitory interneurons has been reported to counter defensive responses (Brandao et al., 2008).

Of note is also a study by Zhang and co-workers reporting systemic administration of TCB-2 to facilitate formation and consolidation of a fear memory, that is, to enhance conditioned freezing but also to facilitate the extinction of a fear memory with reduced freezing as result (Zhang et al., 2013). While several reports suggest 5-HT_2A_ receptors impact memory (Williams et al., 2002; Zhang et al., 2013; Bauer, 2015; Barre et al., 2016), a previous study found no effect of psilocybin (1 or 4 mg/kg) on memory consolidation and merely a modest effect of 4 mg/kg (and no effect of 1 mg/kg) on memory retrieval. That the animals in the present study recognized the context also after psilocybin administration gains support from the observation that the freezing displayed by psilocybin-treated animals, albeit lower than in saline-treated controls, was still considerably higher than that normally displayed by non-conditioned animals in our laboratory (data not shown).

To summarize, the present study adds to the previous literature suggesting 5-HT_2A_ receptors exert a dual impact on fear and anxiety. Dependent on the circumstances, activation of this receptor subtype thus appears both to reduce the expression of conditioned freezing, as evidenced by the anti-freezing effect of 5-HT_2A_ agonists such as psilocybin, and to promote it, as suggested by the anti-freezing effects obtained by adding a 5-HT_2A_ receptor inverse agonist to escitalopram. On the other hand, while being able to impact conditioned freezing when pharmacologically activated—directly by an agonist or indirectly by an SSRI—the 5-HT_2A_ receptor does not seem to be critically involved in such reactions during normal conditions; administered per se the 5-HT_2A_ receptor inverse agonists thus did not influence this response.
